# Retraction Note: Increased biomass productivity in green algae by tuning non-photochemical quenching

**DOI:** 10.1038/s41598-019-48482-w

**Published:** 2019-09-06

**Authors:** Silvia Berteotti, Matteo Ballottari, Roberto Bassi

**Affiliations:** Università di Verona, Dipartimento di Biotecnologie, Strada le Grazie 15, 37134 Verona, Italy

Retraction of: *Scientific Reports* 10.1038/srep21339, published online 18 February 2016

The Authors retract this Article.

Following the publication of this study, we repeated the experiments described in the Article, but could not replicate the results. We were unable to reproduce the strong increase in light use efficiency reported for the npq4 genotype in any condition despite using the same instrumentation and genotypes as in the published study. We have not been able to identify the reasons for the lack of replication and we are retracting the study as we cannot guarantee the accuracy of the conclusions.

MB and RB agree with the retraction of this Article, SB could not be reached.

